# Correction: Optimized Cu-doping in ZnO electro-spun nanofibers for enhanced photovoltaic performance in perovskite solar cells and photocatalytic dye degradation

**DOI:** 10.1039/d4ra90063d

**Published:** 2024-05-31

**Authors:** Kang Hoon Lee, Rabeea Farheen, Zafar Arshad, Mumtaz Ali, Hamza Hassan, Mubark Alshareef, A. Dahshan, Usama Khalid

**Affiliations:** a Department of Energy and Environment Engineering, The Catholic University of Korea 43-Jibong-ro Bucheon-si 14662 Republic of Korea; b Department of Physics, Government College Women University Faisalabad Pakistan; c School of Engineering and Technology, National Textile University Faisalabad 37640 Pakistan zafarnubii@gmail.com; d Department of Chemical Engineering, University of Engineering and Technology Peshawar Pakistan; e Department of Chemistry, Faculty of Applied Science, Umm Al Qura University Makkah 24230 Saudi Arabia mmshreef@uqu.edu.sa; f Department of Physics, College of Science, King Khalid University Abha Saudi Arabia; g Department of Organic and Nano Engineering, Hanyang University 222 Wangsimni-ro, Seongdong-gu Seoul 04763 Republic of Korea

## Abstract

Correction for ‘Optimized Cu-doping in ZnO electro-spun nanofibers for enhanced photovoltaic performance in perovskite solar cells and photocatalytic dye degradation’ by Kang Hoon Lee *et al.*, *RSC Adv.*, 2024, **14**, 15391–15407, https://doi.org/10.1039/D4RA01544D.

The authors regret that one of the affiliations (affiliation *a*) was incorrectly shown in the original manuscript. The corrected list of affiliations is as shown here.

The Royal Society of Chemistry apologises for these errors and any consequent inconvenience to authors and readers.

## Supplementary Material

